# The Arginine/Lysine-Rich Element within the DNA-Binding Domain Is Essential for Nuclear Localization and Function of the Intracellular Pathogen Resistance 1

**DOI:** 10.1371/journal.pone.0162832

**Published:** 2016-09-13

**Authors:** Kezhen Yao, Yongyan Wu, Qi Chen, Zihan Zhang, Xin Chen, Yong Zhang

**Affiliations:** 1 College of Veterinary Medicine, Northwest A&F University, Yangling, Shaanxi, China; 2 Key Laboratory of Animal Biotechnology, Ministry of Agriculture, Northwest A&F University, Yangling, Shaanxi, China; University of Toronto, CANADA

## Abstract

The mouse intracellular pathogen resistance 1 (Ipr1) gene plays important roles in mediating host immunity and previous work showed that it enhances macrophage apoptosis upon mycobacterium infection. However, to date, little is known about the regulation pattern of Ipr1 action. Recent studies have investigated the protein-coding genes and microRNAs regulated by Ipr1 in mouse macrophages, but the structure and the functional motif of the Ipr1 protein have yet to be explored. In this study, we analyzed the domains and functional motif of the Ipr1 protein. The resulting data reveal that Ipr1 protein forms a homodimer and that the Sp100-like domain mediates the targeting of Ipr1 protein to nuclear dots (NDs). Moreover, we found that an Ipr1 mutant lacking the classic nuclear localization signal (cNLS) also translocated into the nuclei, suggesting that the cNLS is not the only factor that directs Ipr1 nuclear localization. Additionally, mechanistic studies revealed that an arginine/lysine-rich element within the DNA-binding domain (SAND domain) is critical for Ipr1 binding to the importin protein receptor NPI-1, demonstrating that this element plays an essential role in mediating the nuclear localization of Ipr1 protein. Furthermore, our results show that this arginine/lysine-rich element contributes to the transcriptional regulation and apoptotic activity of Ipr1. These findings highlight the structural foundations of Ipr1 action and provide new insights into the mechanism of Ipr1-mediated resistance to mycobacterium.

## Introduction

Tuberculosis is a highly infectious disease caused by *Mycobacterium tuberculosis* (*Mtb*). More than 30% of the world population is infected with *Mtb*, but less than a tenth of these individuals is at risk of developing overt clinical symptoms [1], suggesting that the innate immune system plays a crucial role in the defense against *Mtb*. Previous studies found that mouse intracellular pathogen resistance 1 (Ipr1, also known as Speckled 110 KDa protein) mediates innate immunity to *Mtb* and that overexpression of Ipr1 limits bacterial proliferation and reactivates the apoptotic pathway of *Mtb*-infected mouse macrophages [2, 3]. Moreover, polymorphisms of the human homologue of the mouse Ipr1 gene (*Sp110*) are associated with tuberculosis susceptibility [4–6]. Additionally, *SP110* is also as a susceptibility gene for infection with *Mycobacterium avium* subspecies paratuberculosis in cattle [7]. These data reveal the importance of Ipr1 in regulating host immunity to this intracellular pathogen. Therefore, investigating the mechanism of Ipr1-mediated resistance will be useful for improving tuberculosis therapy and for breeding *Mtb*-resistant animals through transgenic technology. To date, however, the molecular mechanism of Ipr1 functions as a potential transcription regulator remains unclear.

Structure is the basis of how proteins perform their functions, and conserved domains or motifs are directly associated with protein function [8]. The mouse Ipr1 protein consists of 455 amino acids and contains a Sp100-like domain and a SAND domain. Previous studies revealed that the Sp100 domain mediates Sp100 protein self-interaction and that the SAND domain is responsible for transcriptional regulation in a series of nuclear proteins [9–14]. Recently, our group reported that Ipr1 has a transcriptional regulatory effect in mouse macrophages in response to *Mtb* infection [3]. However, the detailed regulatory mechanism of how Ipr1 affects downstream genes remains unknown, and the relationship between the protein structure and function of Ipr1 has yet to be explored.

In this study, we characterized the function of the Ipr1 protein domains by combining bioinformatics analyses and experimental studies. We found that the Sp100-like domain is responsible for dimerization and for the targeting of Ipr1 to nuclear dots (NDs). We also mapped two functional classic nuclear localization signals (cNLSs) and validated them by performing functional experiments; however, we found that these two cNLSs are dispensable for Ipr1 nuclear localization. Further studies revealed that a conserved arginine/lysine-rich structural element within the SAND domain not only contributes to Ipr1 nuclear localization as an unconventional nuclear localization signal (NLS), but is also essential for the regulatory effects of Ipr1 on its downstream target genes and signaling pathways.

## Materials and Methods

### Plasmids construction

A variety of full length and truncated mutants sequences were amplified using a vector expressing full-length mouse Ipr1 as template [3], the resulting fragments were cloned into the EGFP-C1, pCMV-HA or p3×FLAG-CMV-10 vectors respectively. The glutathione S-transferase (GST) tag was PCR-amplified from pGEX4T-1 vector, and then ligated into the pEGFP-C1 to generate pEGFP-GST vector. The double-stranded DNA sequence encoding Ipr1 cNLS1 (244–247) and cNLS2 (334–337) were generated by annealing two complementary oligonucleotides, and then inserted into to pEGFP-GST vector. DNA fragments encoding Ipr1 cNLS (244–267 or 334–337) deletion mutants were created by overlap extension PCR. The truncated mutants ΔNLS1/2-434, ΔNLS1/2-423, and ΔNLS1/2-390 were generated by PCR using the both cNLS deleted mutant ΔNLS1/2 as template. The NPI-1 ORF sequence was amplified by PCR from cDNA of RAW264.7 cells. Primer sequences for the plasmids construction were listed in S1 Table. All the constructs were confirmed by DNA sequencing.

### Cell culture and transfection

293FT, RAW264.7 and NIH3T3 cell cells were obtained from the American Type Culture Collection. 293FT and NIH3T3 cells were cultured in DMEM supplemented with 10% fetal bovine serum, and RAW264.7 cells were cultured in RPMI-1640 supplemented with 10% fetal bovine serum. All cells were cultured at 37°C in 5% CO_2_ in humidified incubator. Cells were transfected with FuGENE® HD (Promega, Madison, WI) according to the manufacturer’s specifications.

### Co-immunoprecipitation and Western blotting

Co-immunoprecipitation was carried out using Co-IP Kit (Pierce, Rockford, IL), following manufacturer’s instructions. Immunoprecipitated protein sample were resolved in a 12% SDS-PAGE gel and transferred to a PVDF membrane. Membranes were blocked with 10% non-fat dry milk diluted in TBST for 3h, probed with either anti-FLAG (1:1000 Sigma, Saint Louis, MO), anti-HA (1:1000, Beyotime, Jiangsu, China) or anti-ACTIN (1:2000, Beyotime) antibodies over night at 4°C, and subsequently incubated with HRP-labeled goat anti-mouse antibodies (1:1000, Beyotime). Finally, blots were developed with ECL chemiluminiscence reagent (Beyotime).

### Cross-linking of proteins

293FT cells were lysed in IP lysis buffer (Pierce) for 30 min and the nuclear and cellular debris were cleared by centrifugation. The supernatants were then collected and treated with DMSO alone or 1mM disuccinimidyl suberate (DSS, Pierce) at 37°C for 30 min, then the cross-linker was quenched by adding 1 M Tris-HCL (pH7.5) to a final concentration of 20 mM at room temperature for 15 min. Samples were then solubilized in sample buffer, boiled and centrifuged at 12000 rpm for 5 min.

### Immunofluorescence

Cells were seeded onto 24-well culture plates, grown at 37°C and transiently transfected with indicated construct. Cells expressing proteins tagged with EGFP were fixed with 4% paraformaldehyde for 15 min at room temperature, washed twice with PBS and then stained with DAPI for 15 min to visualize the nuclei. After washing with PBS, cells were visualized by inverted fluorescence microscope. Endogenous PML protein were incubated the anti-PML polyclonal antibody (1:500, Novus, Littleton, CO), diluted in blocking solution, was applied. After incubation at 4°C overnight, the cells were incubated with Alexa555-conjugated goat anti-rabbit antibodies (1:500, Beyotime) diluted in blocking solution for 2 h. After washing twice with PBS and then stained with DAPI for 15 min, and the cells were examined with inverted fluorescence microscope.

### Bioinformatics analysis

Multiple sequence alignments were carried out with Clustal-OMEGA program. The dendrograms were constructed using the DNAman software. The web-based computer software PSORT (http://psort.hgc.jp/) and cNLS mapper (http://nls-mapper.iab.keio.ac.jp/cgi-bin/NLS_Mapper_form.cgi) were used to prediction of potential NLS sequences. Space-filling representation of SAND domain of Ipr1 was done by using the program RASMOL.

### Quantitative PCR

Total RNA was extracted from RAW264.7 cells using Trizol regent (Invitrogen, Carlsbad, CA), and then 1μg of RNA was reverse transcribed to cDNA using SYBR PrimeScript RT reagent Kit (Takara, Dalian, China). The qPCR was performed using SYBR Premix ExTaq II (Takara) on a StepOne Plus PCR system (Applied Biosystems, Foster City, CA). All the primers kept in our laboratory and the specificity of primers has been experimentally test [3]. The comparative CT method was employed for quantification of target mRNA expression.

### Mycobacterial culture and infection

M. tuberculosis strain H37Ra (ATCC 25177) was cultured in Middlebrook 7H9 broth medium supplemented with 10% OADC (Becton, Dickinson and Company, Franklin Lakes, NJ). RAW 264.7 cells were infected at a multiplicity of infection of 5 bacteria per cell (MOI 5:1). After 6 h, washed the infected cells 3 times with RPMI1640 and added new medium for a further 18 h incubation.

### Apoptosis assays

Cells apoptosis was assessed by stained with Alexa Fluor 488-conjugated Annexin V and propidium iodide (PI) (Molecular Probes, Eugene, OR), and then analyzed by flow cytometry (BD Biosciences, San Jose, CA).

### Statistical analysis

All the statistical analysis was represented as the mean ± SD and were analyzed by using the Student’s t-test. A value of p< 0.05 was seen as a sign of significant difference.

## Results

### Homology analysis of Ipr1 protein domains and motifs

**Fig 1:**
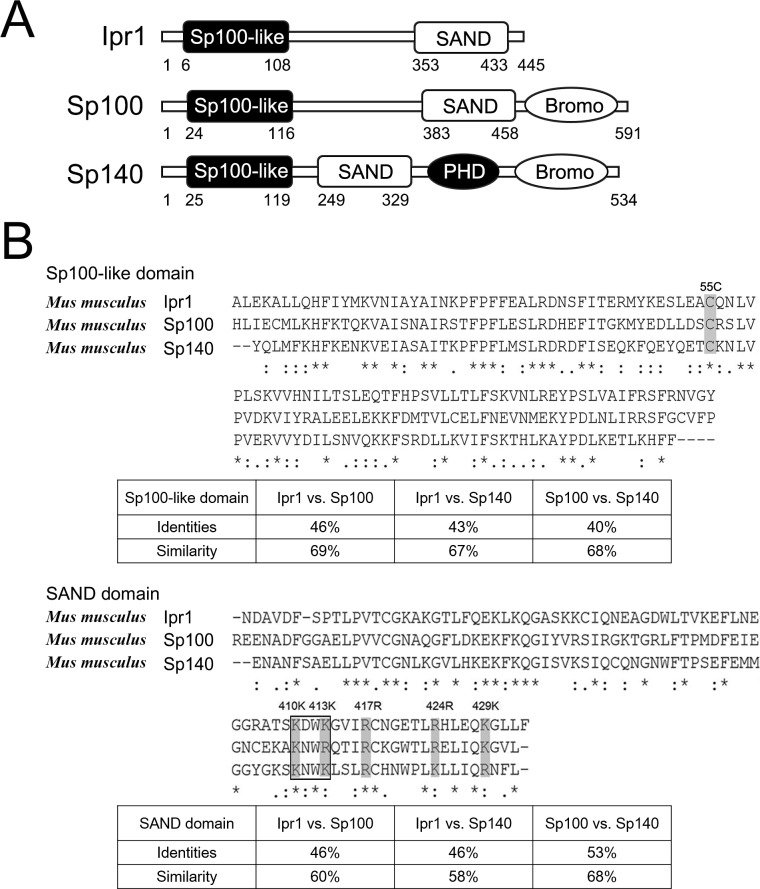
Structure and sequence analysis of Ipr1 protein. (A) Domain structure of Ipr1, Sp100 and Sp140 proteins. Ipr1 protein consists of a conserved Sp100-like domain and a SAND domain containing a DNA-binding motif. (B) Comparison of the amino acid sequences of Sp100-like and SAND domains among Ipr1, Sp100, and Sp140. Identical amino acids are indicated by an asterisk, and strongly or weakly similar amino acids are indicated by a colon or a period. Some of the conserved amino acid residues are marked by shaded boxes. The percentage of identical residues and similarity between domains of Ipr1, Sp100 and Sp140 is calculated in the followed tables.

The sequences of mouse Ipr1 and other proteins were downloaded from the NCBI database, and the conserved domains of Ipr1 protein were analyzed using the Conserved Domain database (http://www.ncbi.nlm.nih.gov/cdd). The Sp100-like domain (aa 6–108) and the SAND domain (aa 353–433) are located on the N-terminus and the C-terminus of the Ipr1 protein, respectively (Fig 1A). The Sp100 domain is involved in nuclear dot (ND)-targeting and in dimerization [9, 14], while the SAND domain is able to mediate DNA binding through a conserved and characteristic KDWK motif (Lys 410–Lys 413 of Ipr1 protein) (Fig 1B) that is part of an emerging group of metazoan transcriptional modulators [11, 13].

Because the promyelocytic leukemia protein (PML)/Sp100 nuclear body family contains both the Sp100-like domain and the SAND domain, we compared Ipr1 with two other Sp100 family members, Sp100 and Sp140 (Fig 1A and 1B). Multiple sequence alignments of each domain were performed by using the program CLUSTAL-OMEGA [15]. The results show relatively high sequence similarity among these three proteins and suggest that some of the core amino acids might be essential for the common functions of these proteins. The highly conserved 55-Cys residues within the dimerization domain (Sp100-like domain) of each monomer might involve in forming interchain disulfide bonds, which generally occurs in homo- and heterodimers protein formation [16]. Moreover, Lys-377, Lys-379, Arg-417, Arg-424, and Lys-429 are highly conserved in the SAND domain, and the basic amino acids close to the KWDK motif have been previously implicated in protein function [10]. Thus, the results of our Ipr1 protein sequence analysis suggest that the Sp100-like domain and the SAND domain might be critical for the function of Ipr1.

### The Sp100-like domain is involved in Ipr1 self-dimerization and ND-targeting

**Fig 2:**
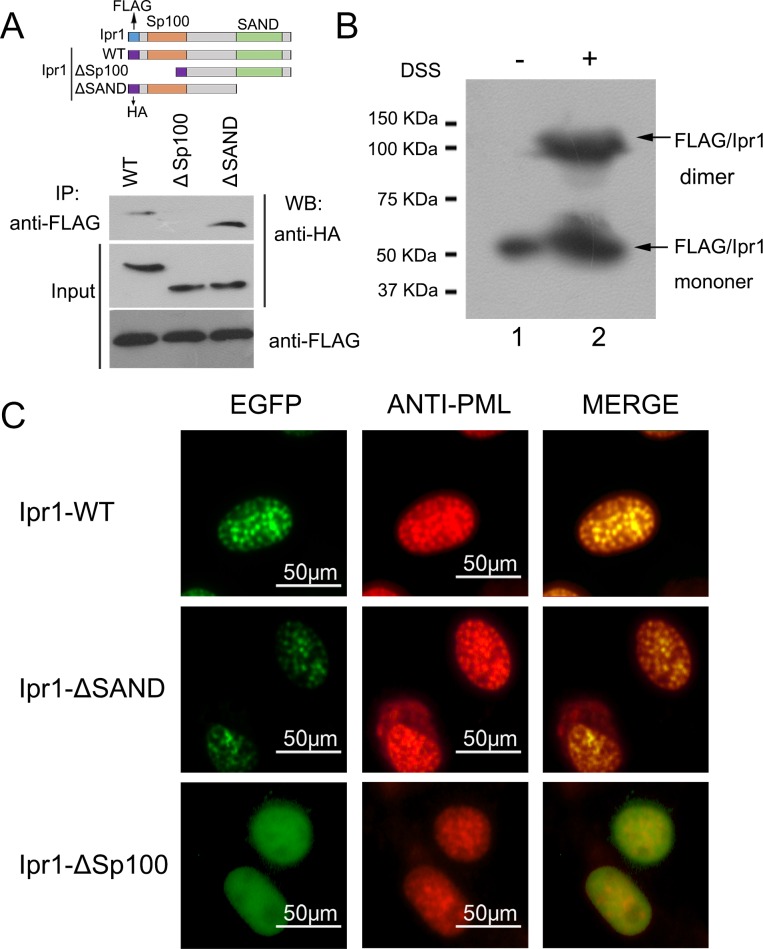
Assessment of the Sp100-like and SAND domain necessities for the dimerization and ND-targeting of Ipr1. (A) Lysates of 293FT cells expressing p3×FLAG-Ipr1 with HA-Ipr1, HA-Ipr1-ΔSp100, or HA-Ipr1-ΔSAND were immunoprecipitated (IP) with anti-FLAG antibodyand detected by western blot (WB) with anti-HA antibody. Input represents 10% of the starting material. (B) 293FT cells were transfected with p3×FLAG-Ipr1. After 24 h, the cells were collected and the resulting cell extracts were subjected to chemical cross-linking by using DSS in DMSO or to DMSO alone as a control. The protein samples were then analyzed by western blot assays using anti-FLAG antibody. (C) RAW264.7 cells were transfected with EGFP-fused Ipr1-WT (top), Ipr1-ΔSAND (middle), or Ipr1-ΔSp100 (bottom). Immunofluorescence was performed using rat anti-PML antibody (red). Merged images show the co-localization of these proteins in yellow.

The results of a two-hybrid assay from a previous study demonstrated that the Sp100 domain in the N-terminus of the Sp100 protein has the potential for homomeric interaction [9]. Given that the Ipr1 protein has a Sp100-like domain with high similarity to the Sp100 protein, we investigated if the Sp100-like domain is responsible for the formation of Ipr1 protein homodimers. 293FT cells were co-transfected with an expression vector encoding FLAG-tagged full-length Ipr1 and an expression vector encoding HA-tagged full-length or truncated Ipr1 (Fig 2A). Cellular extracts were co-immunoprecipitated with anti-FLAG antibody. Interestingly, both HA-tagged full-length Ipr1 and HA-tagged mutant Ipr1 lacking a SAND domain were precipitated with FLAG-tagged Ipr1, but the HA-tagged Ipr1 mutant lacking a Sp100-like domain was not (Fig 2A), indicating that the Sp100-like domain is essential for Ipr1 protein homodimerization. We also noted that the band of HA-tagged full-length Ipr1 from the co-immunoprecipitation experiment was had lower intensity than the band of HA-tagged mutant Ipr1 lacking a SAND domain (Fig 2A), suggesting that the interaction affinity of full-length Ipr1 is lower than that of the SAND domain-deleted mutant Ipr1. This phenomenon might be due to the steric hindrance formed by the structure of the SAND domain.

The above co-immunoprecipitation results suggest the possibility that Ipr1 forms homodimers. To further test the oligomerization state of Ipr1, we used the crosslinking agent disuccinimidyl suberate (DSS), which can crosslink two monomers at adjacent lysine residues to form a stable protein complex. 293FT cells were transfected with a vector encoding FLAG-tagged Ipr1, the total protein collected from these cells was treated with DSS, and these samples were used in western blotting assays performed with anti-FLAG antibody, along with untreated transfected cells, which were included as a negative control. As expected, in addition to detecting a band of approximately 52 KDa, corresponding to the molecular weight of the Ipr1 monomer, we also saw a band with a molecular weight of more than 100 KDa in the DSS-treated sample (Fig 2B). The size of this larger band corresponds to twice the size of monomeric Ipr1, suggesting that it likely contained Ipr1 dimers. In contrast, only one protein band of approximately 52 KDa was detected in the untreated transfected control sample (Fig 2B). Taken together, the results of the co-immunoprecipitation and cross-linking experiments indicate that Ipr1 protein has the ability to form a homodimer.

A previous study showed that the human nuclear body protein Sp110 (homologous to mouse Ipr1) functions as a nuclear hormone receptor transcriptional activator and co-localizes with PML-containing NDs by interacting with Sp140 [17]. To analyze the ND-targeting of mouse Ipr1, mouse macrophage cells, RAW264.7, were transfected with enhanced GFP (EGFP)-fused full-length or truncated Ipr1 expression vectors. The results reveal that full-length and SAND domain-deleted Ipr1 both localized in the nuclei and also co-localized with the PML protein that showed spot-like distribution (Fig 2C). In contrast, Sp100-like domain-deleted Ipr1 showed a diffuse nuclear distribution and was not enriched in the NDs (Fig 2C). These data indicate that the Sp100-like domain mediates the formation of Ipr1 homodimers as well as their nuclei colocalization with NDs.

### Identification of the Ipr1 nuclear localization signal

**Fig 3:**
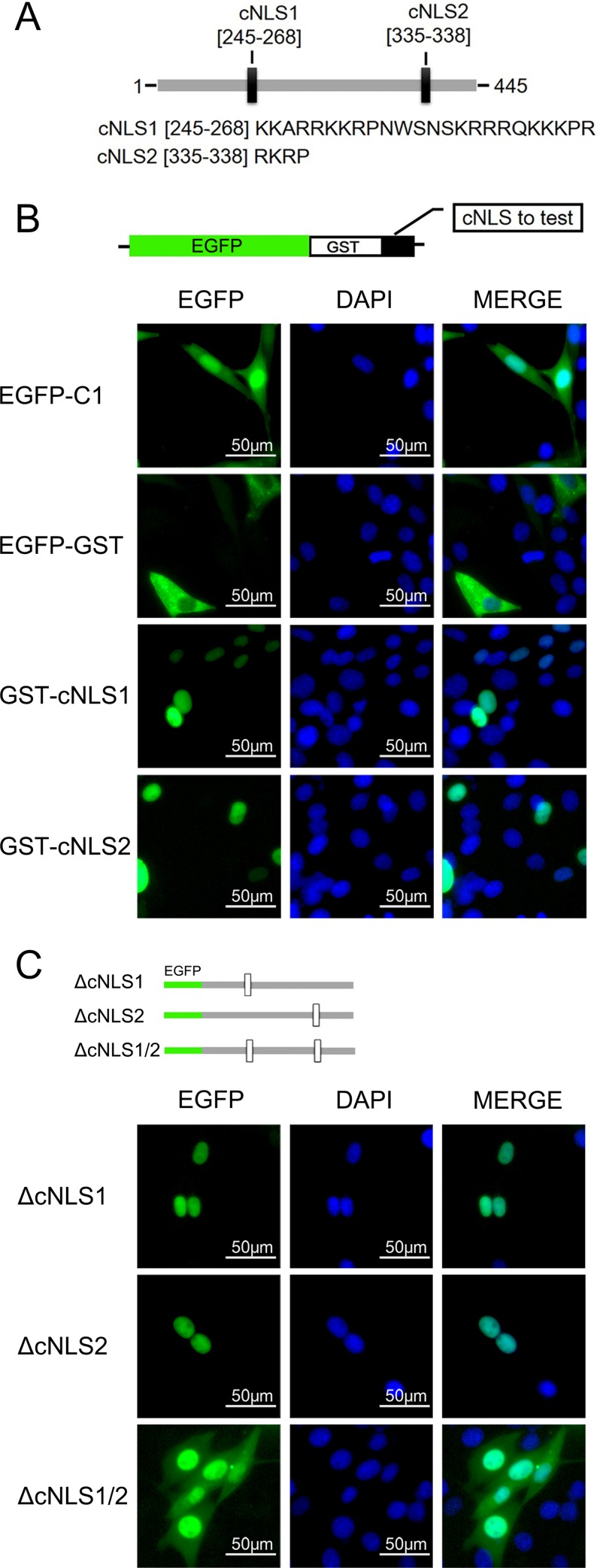
Functional validation of the Ipr1 cNLSs. (A) Schematic representation of Ipr1 protein. The positions of two cNLSs (black rectangles) identified by web-based program analyses are indicated, and the sequence of each cNLS is presented below. (B) Schematic representation of the EGFP-GST construct used in the nuclear import assessment. Each Ipr1 cNLS sequence was cloned into downstream of the EGFP-GST. The fluorescence images show representative samples of NIH3T3 cells transfected with each of the expression plasmids for 24 h. The cell nuclei were counterstained with DAPI. (C) Schematic representation of Ipr1 mutants bearing deletions of cNLS1, cNLS2, or both. The fluorescence images show representative results from the nucleocytoplasmic localization of these Ipr1 mutants in NIH3T3 cells. The cell nuclei were counterstained with DAPI.

Mouse Ipr1 protein contains a predicted nuclear localization signal (NLS) [2]. However, the detailed sequence that mediates Ipr1 import into the nucleus has not been functionally characterized. After verifying the nuclear localization of Ipr1 protein, we aimed to identify the motif that affects Ipr1 nuclear localization. The potential Ipr1 NLS was predicted using web-based software programs, PSORT [18] and NLS mapper [19], and two putative classic nuclear localization signals (cNLSs), designated cNLS1 (aa 244–267) and cNLS2 (aa 334–337) were identified by both of the two programs (Fig 3A). To investigate the importance of these two cNLSs on nuclear localization, the abilities of cNLS1 and cNLS2 to target cytoplasmic protein (EGFP-GST) into the nucleus was determined. As shown in Fig 3B, both cNLS1- and cNLS2-fused EGFP-GST showed fully nuclear localization in NIH3T3 cells, suggesting that both cNLS1 and cNLS2 are functional NLSs.

Next, we generated cNLS-deleted Ipr1-fused EGFP expression vectors to verify the contribution of each cNLS to the nuclear localization of Ipr1 protein. We found that Ipr1 lacking either cNLS1 or cNLS2 had a similar nuclear localization as the wildtype Ipr1, which indicates that the deletion of either cNLS1 or cNLS2 alone does not alter the subcellular localization of Ipr1 (Fig 3C). Unexpectedly, the Ipr1 mutant lacking both cNLS1 and cNLS2 was mainly distributed in the nuclei (Fig 3C). Given that the molecular size of the EGFP-fused Ipr1 proteins (75–80 KDa) was much bigger than the size limit (40–60 KDa) of the nuclear pore complex [20], we speculated that some atypical mechanism of nuclear transport mediates Ipr1 nuclear translocation and that factors other than cNLSs may affect Ipr1 nuclear localization. Taken together, these results reveal that the cNLSs of Ipr1 contribute to the nuclear localization of Ipr1, but that there must be other more important motif(s) and/or regulator(s) that direct Ipr1 subcellular localization.

### An arginine/lysine-rich structural element in the Ipr1 C-terminus of is required for its nuclear localization

**Fig 4:**
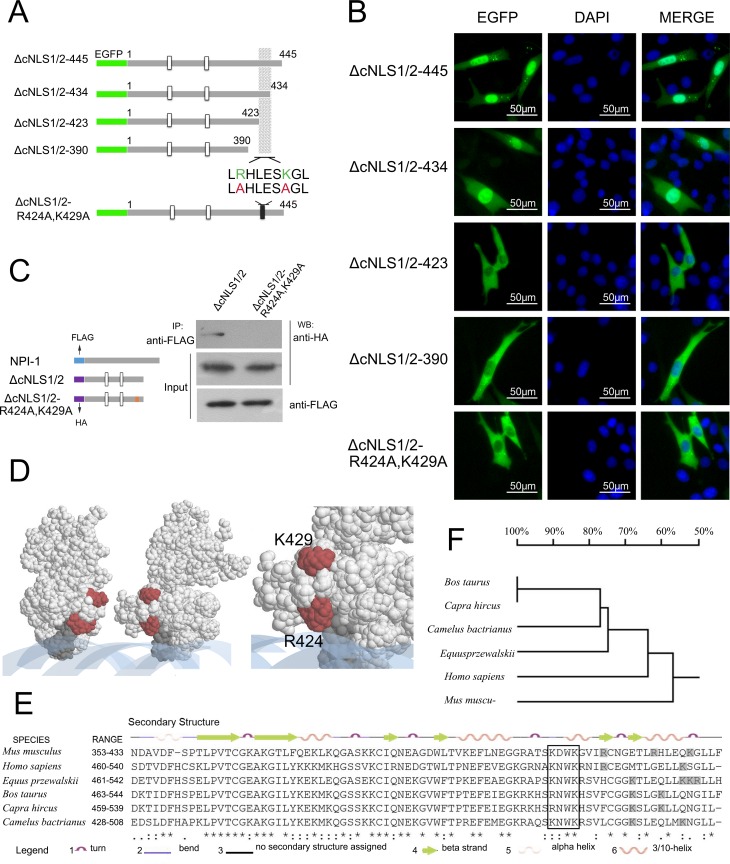
Assessment of the Ipr1 arginine/lysine-rich element as a non-classical NLS of Ipr1. (A) Schematic illustrations of the different EGFP-fused constructs with different C-terminus deletion constructs of Ipr1-ΔNLS1/2. The minimal motif thought to contribute to nuclear import is shaded. The double substitutions (red) of key basic residues (green), Arg-424 and Lys-429, were shown at the bottom. (B) The fluorescence images show representative results of NIH3T3 cells transfected with each plasmid shown in (A) for 24 h. The cell nuclei were counterstained with DAPI. (C) An NPI-1 interaction test was performed with lysates from transiently co-transfected 293FT cells expressing p3×FLAG-NPI-1 with ΔcNLS1/2 or ΔcNLS1/2-R424A-K429A. These lysates were immunoprecipitated (IP) with anti-FLAG antibody (Sigma), and the interactions were detected by performing western blots (WB) with anti-HA antibody. Input represents 10% of the starting material. (D) A space-filling representation of the Ipr1 monomer SAND domain was obtained by using the program RASMOL (Rutgers Protein Data Bank accession number: 1ufn). The KDWK motif is shown in dark grey, DNA in blue, and the amino acids involved in the arginine/lysine rich element in red. The enlargement shows the amino acids comprising the arginine/lysine-rich elements. (E) Multiple sequence alignment of the mouse Ipr1 protein sequence with homologous proteins from humans, cattle, goats, horses, and camels. Secondary structure elements are shown on top of the alignment. The KDWK motif is indicated in a white box, and the arginine/lysine residues adjacent to the KWDK motif are shown in shaded boxes. (F) The dendrogram shows the evolutionary relationships of the Ipr1 SAND domains between different species.

To further identify the motif that determines Ipr1 nuclear localization, we investigated the role of non-classic NLSs in mediating Ipr1 nuclear localization. Because NLSs immediately adjacent to the DNA-binding domain or overlapping with the DNA-binding domain have been reported in the majority of DNA-binding proteins [21], we generated EGFP-fused Ipr1 expression constructs with truncated SAND domains based on the Ipr1 mutants lacking both cNLSs, ΔcNLS1/2 (Fig 4A). In NIH3T3 cells, the Ipr1 mutant with a deletion of aa435–445 exhibited a similar subcellular localization to that of full-length Ipr1 (Fig 4B). Remarkably, mutants lacking aa424–445 or aa391–445 localized exclusively to the cytoplasm (Fig 4B), indicating that a non-classic NLS exists within the region between Ipr1 protein aa423 and aa435. This region consists of a cluster of basic amino acids near the KDWK motif that are conserved in Ipr1, Sp100, and Sp140 proteins (Fig 1B) and includes amino acids Arg-424 and Lys-429. To investigate the effect of Arg-424 and Lys-429 on Ipr1 nuclear localization, we generated EGFP-fused Ipr1 mutants containing mutations in both Arg-424 and Lys-429 (ΔNLS1/2-R424A-K429A) (Fig 4A). The results reveal that mutations of Arg-424 and Lys-429 led to the cytoplasmic localization of Ipr1 (Fig 4B).

Because the arginine/lysine-rich motif was found to be responsible for Ipr1 nuclear localization and the importin protein receptor importin-α5 (NPI-1) mediates protein translocation into the nuclei [22, 23], we investigated the interaction between the Ipr1 mutants ΔcNLS1/2 or ΔcNLS1/2-R424A-K429A and NPI-1 (Fig 4C). Co-immunoprecipitation experiment results show that NPI-1 binds to Ipr1-ΔcNLS1/2 mutant, but not to the ΔcNLS1/2-R424A-K429A mutant, indicating that the Arg-424 and Lys-429 in the SAND domain contribute to the interaction between Ipr1 and NPI-1, thus mediating Ipr1 nuclear localization.

The 3D configuration of the Ipr1 protein SAND domain was modeled using RASMOL [24], and the resulting space-filling representation is shown in Fig 4D. This partial structure of Ipr1 protein suggests the possibility that when two monomers move close to each other, the adjacent arginine/lysine-rich structural elements are exposed to the surface, thus providing the chance for recognition by the importin receptor protein.

Additionally, the alignment of Ipr1 with its orthologous proteins in other species shows that a conserved arginine/lysine-rich structural element exists in the SAND domain, but the specific site varies following the evolution and relationships among species (Fig 4E and 4F).

Collectively, these data indicate that an arginine/lysine-rich structural element in the SAND domain is required for Ipr1 nuclear localization, and this region is essential for the interaction between Ipr1 and the importin receptor protein NPI-1.

### Arg-424 and Lys-429 are critical for Ipr1 function

**Fig 5:**
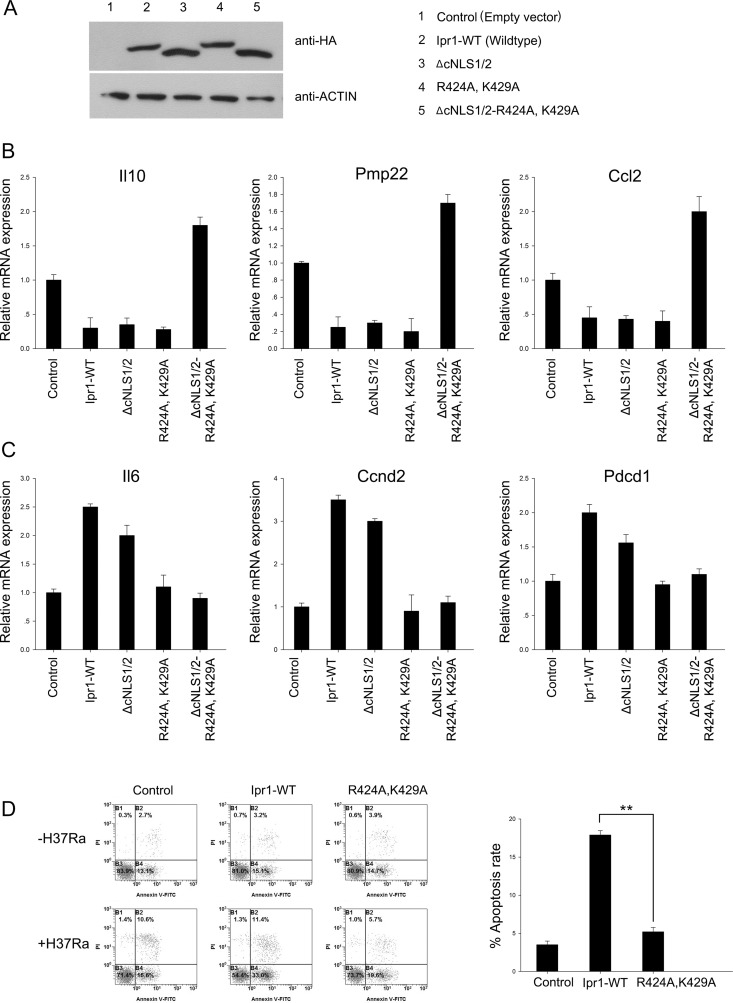
Functional activity of wildtype and mutant Ipr1 in regulation assays. (A) A representative blot from a western blot analysis demonstrating the expression of the different mutant proteins. (B) Results from an assay measuring the gene repression induced by Ipr1. The expressions of Il10, Pmp2, and Ccl2 were determined by qPCR after the transient transfection of RAW264.7 cells with wildtype Ipr1 or various Ipr1 mutants. (C) Results from an assay measuring the gene activation induced by Ipr1. The expressions of Il6, Ccnd2, and Pdcd2 were determined by qPCR after the transient transfection of RAW264.7 cells with wildtype Ipr1 or various Ipr1 mutants. (D) RAW 264.7 cells were transfected with empty vector as control, wildtype Ipr1, or Ipr1-R424A-K429A. After 12 h, each group of transfected cells was incubated in the absence or presence of H37Ra. Apoptotic cells were evaluated by Annexin-V staining followed by flow cytometric analysis. The apoptotic cell rate, which is presented in the right panel, was quantified by the following algorithm: percentage of Annexin-V+ and PI− cells in the presence of H37Ra minus the percentage of Annexin-V+ and PI− cells in the absence of H37Ra. Data represent the mean ± SD of three independent experiments. Two asterisks, p < 0.01.

Our recent study revealed that Ipr1 regulates the transcription of cytokines, chemokines, and genes involved in cell death and in the intracellular survival of *Mtb* [3]. Because the Arg-424 and Lys-429 of Ipr1 that regulate its nuclear localization are very close to the Ipr1–DNA interaction site, we tested whether or not these amino acids are associated with the transcriptional regulation and apoptosis that are mediated by Ipr1. RAW264.7 cells were transfected with a series of Ipr1 mutant constructs. At 36 h post-transfection, the protein levels of wildtype and mutated Ipr1 were evaluated by western blotting (Fig 5A), and the expressions of several genes previously identified as being downstream of Ipr1 were determined by quantitative (q) PCR, including Ccl2, Ccnd2, Il6, Il10, Pdcd1, and Pmp22 [3]. The resulting data confirm previous results that Ipr1 inhibits Il10, Pmp22, and Ccl2. Moreover, the deletion of cNLS1/2 or the mutation of Arg-424/Lys-429 alone did not significantly alter the suppression by Ipr1 of Il10, Pmp22, and Ccl2 (Fig 5B). Notably, the simultaneous deletion of cNLS1/2 and mutation of Arg-424/Lys-429 abolished the inhibitory effects of Ipr1 on Il10, Pmp22, and Ccl2, resulting in an upregulation of these genes (Fig 5B). Additionally, both the wildtype and the cNLS1/2-deleted Ipr1 upregulated the genes Il6, Ccnd2 and Pdcd1, while the Ipr1 with mutated Arg-424 and Lys-429 was unable to upregulate these genes (Fig 5C). These results suggest that Arg-424 and Lys-429 are associated with the transcriptional regulation of Ipr1, especially the transcriptional activation.

Next, we analyzed the apoptotic rates of RAW264.7 cells overexpressing the wildtype Ipr1 or the Arg-424/Lys-429 mutant Ipr1 following H37Ra infection. The results show that although RAW264.7 cells are not sensitive to H37Ra-induced apoptosis, the overexpression of wildtype Ipr1 dramatically increased macrophage apoptosis. In contrast, the RAW264.7 cells overexpressing Arg-424/Lys-429 mutated Ipr1 exhibited lower apoptotic rates after H37Ra infection than the cells transfected with wildtype Ipr1 (Fig 5D). Collectively, these data indicate that the Arg-424 and Lys-429 of Ipr1 are critical for its biological functions.

## Discussion

The Ipr1 protein, a member of the Sp100/Sp140 family, is strongly induced by type II interferon-γ [25], and expression of Ipr1 in mice improved their resistance to *Mtb* infection [2]. Additionally, Sp110 nuclear body protein, the human homologue of mouse Ipr1, plays important roles in the pathogenesis of infectious disease; Sp110 protein can facilitate the infection or replication of *Anaplasma phagocytophilum* and Epstein-Barr virus [26, 27]. Furthermore, mutations in the Sp110 gene have been associated with immunodeficiency diseases, such as viral hepatitis infection [28] and hepatitis C virus infection-induced chronic liver diseases [29]. Recent studies found that Sp110 polymorphisms are linked to tuberculosis susceptibility [5, 30]. Our previous study showed that overexpression of mouse Ipr1 enhances host cell resistance to virulent strains of *Mycobacterium bovis* both *in vitro* and *in vivo* [31, 32]. Additionally, we used high-throughput sequencing to investigate the downstream genes involved in the Ipr1-mediated network, and the results suggest that Ipr1 protein regulates innate immunity and apoptosis in response to *Mtb* infection via modulating the expressions of a series of genes [3].

To better understand the transcriptional regulation function of Ipr1, we analyzed and characterized its functional domains and their effects on Ipr1 dimerization, ND targeting, and nuclear localization. In addition to cNLSs, we found that an arginine/lysine-rich element in the SAND domain plays an essential role in regulating Ipr1 nuclear localization. Furthermore, we found that mutations in the Ipr1 Arg-424 and Lys-429 abolish the transcriptional regulation activity of Ipr1 and affect Ipr1-mediated apoptosis in *Mtb*-infected macrophages.

The protein sequence and domains of Ipr1 are similar to those of the nuclear body proteins Sp100 and Sp140, suggesting an inherent correlation among these three proteins. It has been reported that Sp140 enhances the localization of Sp110 to the PML-Sp100 nuclear body [17]. Our data provide evidence that Ipr1 can form a homodimer through the interaction of its Sp100-like domain, which is also responsible for its ND targeting. The dimerization domains of the Sp100 family proteins are highly conserved, raising the possibility that Ipr1 forms a heterodimer with either Sp100 or Sp140. Transcription factors bind to specific DNA elements by forming a homodimer or a heterodimer, especially for nuclear proteins with a SAND domain, such as NUDR, AIRE-1, and GMEB [10–13]. Matthew et al. defined a novel DNA-binding fold in SAND in which a conserved positively-charged surface patch is found within an α-helix located in the KDWK sequence motif [10], and similar secondary structure characteristics were found on Ipr1 by DSSP analysis (Fig 4E). The DNA-binding region in the structure of the Sp100 protein SAND domain has been mapped by NMR spectrum, and the resulting sequence, 5ʹ-CCTTGCGCAAGG-3ʹ, is considered to be a possible ligand [10]. Sp110 was also shown to modulate gene transcription by binding to the gene promoter region [17], and further studies are needed to identify the sequence specificity for Ipr1 binding to DNA, thus elucidating the detailed role of Ipr1 in transcriptional regulation.

The subcellular location of nuclear proteins is closely related to their function. Aberrant subcellular localization of Sp110b due to virus infection resulted in the inhibition of RAR alpha-mediated transcription [29]. Hence, understanding the mechanism for Ipr1 nuclear localization is critical for unraveling Ipr1-mediated resistance to *Mtb*. Bloch et al. speculated that there is a NLS in human Sp110 protein between amino acids 288 and 306 [17]. Additionally, Li et al. recently found that a lack of Sp110 amino acids 251–280 in pigs decreased Sp10 nuclear accumulation [33]. In most cases, the subcellular location of a nuclear protein is mediated by a NLS. However, our results indicate that Ipr1 nuclear localization is controlled by something other than this classic mechanism. Although, we identified two Ipr1 cNLSs that are sufficient to translocate a reporter protein (EGFP-GST) into the nucleus, the deletion of these cNLSs did not completely abolish the Ipr1 nuclear localization, indicating that other factors regulate Ipr1 nuclear localization.

**Fig 6:**
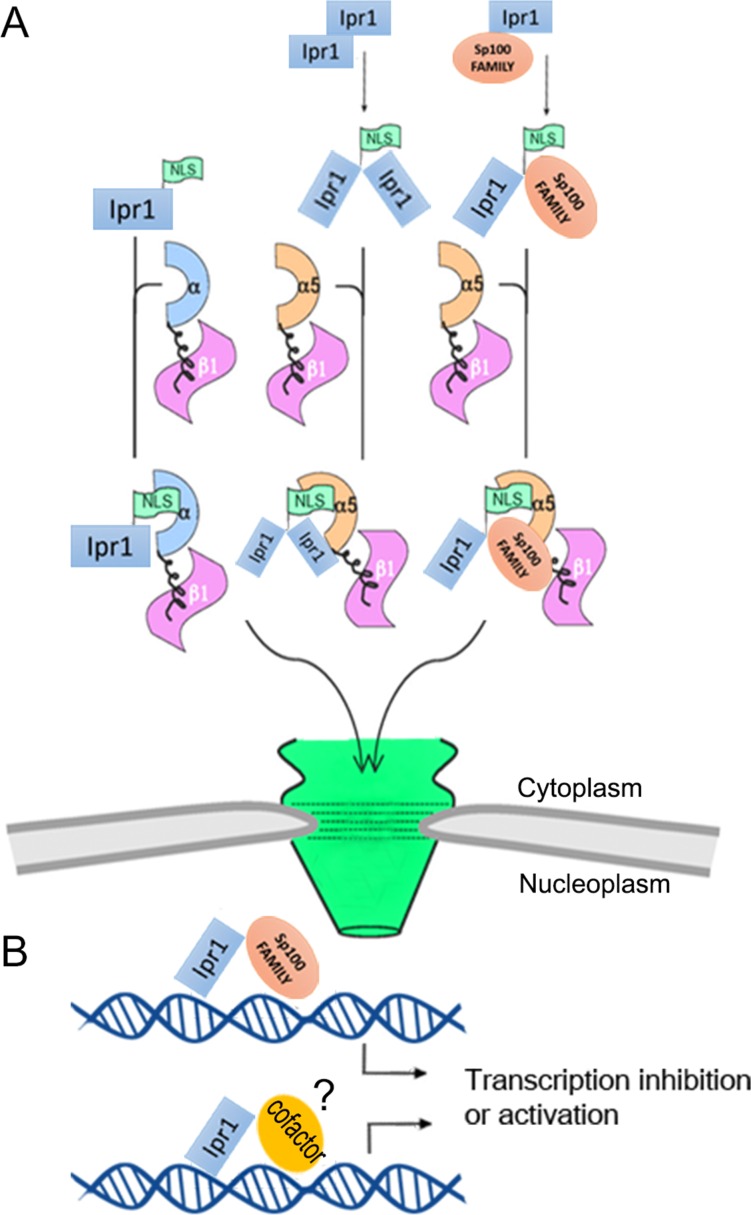
Schematic representation of the potential mechanism for Ipr1 nuclear import and transcriptional regulation. (A) A model illustrating the ability of Ipr1 forms homo/hetero dimers and transports into the nuclear by binding with importin protein. (B) The hypothesized regulation mode of Ipr1 on both transcriptional inhibition and activation.

In approximately 80% of transcription factors or nuclear proteins, the NLS overlaps or is immediately adjacent to the DNA-binding domain [21]. Our sequence analysis of Ipr1 protein revealed an arginine/lysine-rich element in the DNA-binding domain (the SAND domain). This element is responsible for the retention of Ipr1 protein in the nucleus. Our data also demonstrate that the Arg-424 and Lys-429 in the Ipr1 SAND domain function as a conformational NLS, which is essential for the interaction between Ipr1 and NPI-1 that is needed to translocate Ipr1 into the nucleus. A dimer-specific NLS (ds-NLS) in STAT dimers has been mapped to the STAT DNA-binding domain [34], and it contains two basic amino acids separated by several residues (KXnKXnR) that are only functional when binding with NPI-1 following STAT dimerization [23, 35]. Ipr1 has an arginine/lysine-rich element, which is similar to that of STAT and also interacts with NPI-1. We found that the non-classic NLS in Ipr1 is partially responsible for its nuclear localization. Hence, we hypothesized that the dimer state of Ipr1 generates a new NLS that can translocate the dimer protein into the nuclei (Fig 6A). Further studies of Ipr1 mutants that lack the ability to form dimers are required to gain a better understanding of the significance of dimerization for Ipr1 nuclear localization.

Recently, our group reported that Ipr1 regulates mRNA and miRNA expression in mouse macrophages in response to *Mtb* infection [3]. The Arg-424 and Lys-429 in Ipr1 are close to the Ipr1 DNA-binding domain and affect its nuclear localization, so we speculated that Ipr1 Arg-424 and Lys-429 might play roles in the transcriptional regulation function of Ipr1. Surprisingly, the results show that mutations of the Arg-424 and Lys-429 did not affect Ipr1-dependent repression; instead, they resulted in a decrease of transcriptional activation. Previous studies have reported mutations in the basic amino acids adjacent to the KDWK motif of another SAND domain containing nuclear protein, NUDR, did not affect its transcriptional repression but instead resulted in its reduced transcriptional activation [10]. This indicates that the mechanisms for Ipr1 SAND domain-mediated regulation of transcriptional activation or repression may be different. It also suggests that this Arg-424 and Lys-429 conserved element plays pivotal roles during the regulation of transactivation, such as its interaction with other cofactors (Fig 6B). The lack of this inhibitory effect in the mutant ΔcNLS1/2-R424A-K429A is probably due to it forming a dimer with endogenous Ipr1, thus sequestering endogenous Ipr1 from the nucleus. Moreover, the results of our apoptosis assays demonstrate that Ipr1 Arg-424 and Lys-429 confer apoptosis activity induction by *Mtb*. Collectively, our mutagenesis data imply that Arg-424 and Lys-429 in Ipr1 are required for its transcriptional activation and H37Ra-mediated apoptosis induction.

In summary, we comprehensively investigated the function of Ipr1 domains by combining bioinformatics analyses and experimental studies. The features of Ipr1 ND targeting, dimerization, and nuclear localization were mapped to domains or motifs. Moreover, the data reveal that the transcriptional regulation by Ipr1 on downstream genes and apoptosis activity are directly related to the Arg-424 and Lys-429 in the SAND domain. These findings provide new insights into the protein structure of Ipr1, laying the foundations for elucidating the mechanism of Ipr1-mediated macrophage resistance to *Mtb*. The Ipr1 arginine/lysine element identified here might potentially be targeted to disrupt the subcellular location or the gene expression for therapeutic purposes. Additionally, our findings provide the theoretical basis for using ChIP-seq assays or mass spectrometry techniques to further investigate the specific DNA-binding element or to test the interaction of Ipr1 with co-activators.

## Supporting Information

S1 Table Primer sequences for plasmids construction. Sequences of the primers used for the construction of all plasmids used in this study.
(PDF)
