# Supplementary material for: The Arginine/Lysine-Rich Element within the DNA-Binding Domain Is Essential for Nuclear Localization and Function of the Intracellular Pathogen Resistance 1
Source: PLoS One. 2016 Sep 13;11(9):e0162832. doi: 10.1371/journal.pone.0162832 (PMC5021326; doi:10.1371/journal.pone.0162832)
Supplement: S1 Table — Sequences of the primers used for the construction of all plasmids used in this study. (PDF) [file pone.0162832.s001.pdf]

Primer sequence	Forward	Reverse
Co-immunoprecipitation		
p3XFLAG-Ipr1-wt	CGC**GAATTC**AATGTTCACTCTGACCAAAGCCT	ACT**GGTACC**GACTAGGCACCCTTCTTTTGAGG
HA-Ipr1	CC**GAATTC**GGATGTTCACTCTGACCAAAGCCTTG	CGC**GGTACC**CTAGGCACCCTTCTTTTGAGG
HA-Ipr1-ΔSP100	CC**GAATTC**GGTATACCTACGAAGAGAAAAACAG	CGC**GGTACC**CTAGGCACCCTTCTTTTGAGG
HA-Ipr1-ΔSAND	CC**GAATTC**GGATGTTCACTCTGACCAAAGCCTTG	CGC**GGTACC**CTAAGAGAAATCCACAGCGTCATT
p3XFLAG-NPI-1	CGC**GAATTC**AATGTCCACACCAGGAAAAGAGAACTT	ACT**GGTACC**GATCAAAGCTGGAAACCTTCCATAGGA
Fluorescence (GFP-C1)		
Ipr1	TTC**GAATTC**TATGTTCACTCTGACCAAAGCCTTG	CGC**GGTACC**CTAGGCACCCTTCTTTTGAGG
Ipr1-108	TTC**GAATTC**TTATACCTACGAAGAGAAAAACAG	CGC**GGTACC**CTAGGCACCCTTCTTTTGAGG
Ipr1-353	TTC**GAATTC**TATGTTCACTCTGACCAAAGCCTTG	CGC**GGTACC**CTAAGAGAAATCCACAGCGTCATT
GST	CTC**AGATCT**ATGTCCCCTATACTAGGTTATTGGA	TCG**AAGCTT**GTCACGATGCGGCCGCTCGAGTCGA
NLS1/2-435	TTC**GAATTC**TATGTTCACTCTGACCAAAGCCTTG	CGC**GGTACC**CTAAAAGGACTTTTGTTCTTT
NLS1/2-423	TTC**GAATTC**TATGTTCACTCTGACCAAAGCCTTG	CGC**GGTACC**CTATAATGTCTCCCCGTTACAACGTAT
NLS1/2-390	TTC**GAATTC**TATGTTCACTCTGACCAAAGCCTTG	CGC**GGTACC**CTACTCATTCTGAATGCACTTTTTTGA
NLS1/2-mut	TTC**GAATTC**TATGTTCACTCTGACCAAAGCCTTG	CGC**GGTACC**CTAGGCACCCTTCTTTTGAGGTTTACTCTTGGAGGTAAAGAACAAAAGTCCGGCCTGCTCCAGATGGGCTAATGTCTCCCCGTTACAACGTA
Two steps PCR		
ΔcNLS1	TTC**GAATTC**TATGTTCACTCTGACCAAAGCCTTG	TCCCATCATCTCATCTTGGTTTGCAGGTGTGCAGGG
	CCCTGCACACCTGCAAACCAAGATGAGATGATGGGA	CGC**GGTACC**CTAGGCACCCTTCTTTTGAGG
ΔcNLS2	TTC**GAATTC**TATGTTCACTCTGACCAAAGCCTTG	GTTCTTCGTGCTGTGCTACCACTTGTTTTTGATG
	CATCAAAAACAAGTGGTAGCACAGCACGAAGAAC	CGC**GGTACC**CTAGGCACCCTTCTTTTGAGG

S1 Table. Primer sequences for plasmids construction. Sequences of the primers used for the construction of all plasmids used in this study.
